# Burnout rate among dental professionals post COVID-19 at one academic dental institution

**DOI:** 10.1186/s12909-025-07841-0

**Published:** 2025-09-25

**Authors:** Utsavi H. Kapadia, Sanam Akhavan, Manwa M. Hegde, Jaspreet Singh Cheema, Keerthini Veluru, Alireza Karimi, Nazgol Zamanian, Elbert Tom, Sohail Ebrahimi, Ram M. Vaderhobli

**Affiliations:** 1 Department of Preventive and Restorative Dental Sciences (PRDS), UCSF School of Dentistry, San Francisco, USA; 2 UCSF School of Dentistry, Los Angeles, USA; 3 Department of Preventive and Restorative Dental Sciences (PRDS), UCSF School of Dentistry, Director AEGD NYU Langone, 707 Parnassus Ave, Suite 4000, San Francisco, CA 94143 USA

**Keywords:** COVID-19, Burnout, Mental health, Professionals, Stress, Anxiety, Depression

## Abstract

**Objective:**

The COVID-19 pandemic has affected the healthcare profession worldwide. Frontline healthcare workers have experienced increased personal and occupational stress related to burnout, depression, and anxiety. The long-term effects of burnout, anxiety and mental suffering may lead to social isolation, substance abuse and behavioral changes. The goal of this study is to assess the self-reported levels of burnout and institutional support among dental students, residents, faculty and staff at the University of California, San Francisco in the aftermath of the COVID-19 pandemic.

**Methods:**

A 15-minutes short survey questionnaire was distributed to all dental students, dental residents, dental faculty, and staff through Qualtrics^®^ online survey tool. Out of 109 participants, 108 individuals completed the survey during September 2023. This study was approved by the university’s institutional review board (IRB # 23-39814). After demographic information, institutional support information was collected, the Copenhagen Burnout Inventory (CBI) was used to assess personal and work-related burnout.

**Results:**

During the period of the study there were 109 participants. We found that most professionals rated institutional support as Good. The CBI subscale scores revealed that both personal and work-related burnout were prevalent among study participants. A total of 108 dental professionals at UCSF completed a 21-item questionnaire assessing the impact of COVID-19 on wellbeing and burnout, with 55.56% (*n* = 60) reporting changes in their work-related wellbeing. Based on the Copenhagen Burnout Inventory, 37.74% (*n* = 40) reported moderate, 8.49% (*n* = 9) high, and 1.89% (*n* = 2) severe personal burnout, while 38.68% (*n* = 41) experienced moderate and 14.15% (*n* = 15) high or severe work-related burnout.

**Conclusion:**

Our study findings emphasize the need for proactive measures to address burnout and support the well-being of dental students, faculty, and staff.

**Practical implications:**

The findings suggest that dental institutions should implement targeted support systems and mental health resources to address burnout among dental professionals effectively. By fostering a supportive environment, institutions can improve both professional well-being and patient care quality.

**Supplementary Information:**

The online version contains supplementary material available at 10.1186/s12909-025-07841-0.

## Introduction

In May 2023, the coronavirus disease-19 (COVID-19) was no longer considered a worldwide health emergency [1]. However, public health agencies remain concerned about the disease, with 767 million confirmed cases and 6.9 million deaths to date [1]. Despite the gradual lifting of restrictions due to the development of vaccines and anti-virus medications, healthcare professionals continue to battle regional spikes in cases and fatalities.

Burnout, a condition characterized by depersonalization (DP), emotional exhaustion (EE), and a decreased sense of personal accomplishment (PA), is a result of workplace factors rather than individual traits [1]. Burnout is a problem with the working environment and not with the individual. The layout and operation of the workplace have an influence on how employees interact with each other and go about accomplishing their work performance [2]. The COVID-19 pandemic forced a redesign of organizational networks and considerable changes in practitioners’ personal and professional lives; therefore, it has been demanding on healthcare organizations and professionals [3]. Workplace culture, occupational stress, and workload pressures are known contributing factors to burnout [4], and these factors have worsened in the current environment [5]. 

The long-term effects of burnout include anxiety, rage, sadness, post-traumatic stress disorder symptoms, substance abuse, and behavioral adjustments such as avoiding social gatherings and practicing cautious hand hygiene [6]. The COVID-19 pandemic has also impacted the protective effect of emotional intelligence on burnout and wellbeing among healthcare personnel [7]. 

During a pandemic, effectively coordinating medical supplies, treatment facilities, and the workforce is crucial and affects the burnout rate [8]. As the depth of knowledge about the connection between job burnout and occupational stress has grown, some researchers have discovered that occupational stress influences job burnout indirectly through other characteristics, such as social support, in addition to having a direct impact on it [9]. Employees’ possibility of leaving their jobs voluntarily in the near future is known as their turnover intention, which can refer to either internal or external intentions. People leaving an organization are considered external turnover (ET), whereas transfers within an organization, including requests to switch departments, are considered internal turnover (IT) [10]. It is evident through our scores that surveys and assessments can assist establishments to stay proactive in addressing burnout by identifying predictors and implementing targeted interventions. By doing this, establishments can alleviate the effects of burnout, which ultimately will enhance the quality of patient care in all healthcare institutions. Healthcare systems are concerned about turnover because it is linked to inferior performance on both the individual and organizational levels, a major loss in care quality, an increase in workload for the employees who stayed, a drop in morale, and more turnover. Especially during the COVID-19 period, turnover was identified as one of the factors impeding the development of the healthcare system [10]. 

According to previous surveys, working in dentistry is becoming exhausting. Patient satisfaction is likely to be low when visiting burned-out healthcare professionals [11]. Loss of professional control, autonomy, and flexibility; ineffective procedures; conflicting workplace relationships and goals; excessive administrative tasks; dissatisfaction with medical record systems; and a lack of work-life balance are some of the factors linked to burnout [12]. Altman et al. (2023) highlighted how limited workplace support and structural pressures affect employee well-being in general settings. It is a pattern that closely resembles dental practice. In dentistry, stressors such as time pressure, prolonged procedures, confined workspaces, and the pursuit of technical perfection contribute to increased mental health risks among providers and staff [13]. 

The worldwide need for public health resources and infection control measures has increased due to the COVID-19 pandemic. Investigating occupational stress and psychological well-being is crucial to minimize the detrimental effects of pandemic-related occupational challenges. It is also essential to find suitable resource management [14]. The present COVID-19 pandemic has increased the occupational stress experienced by dental professionals during the past two years [15]. The pressure and stress that COVID-19 places on healthcare professionals, such as physicians and dentists, leading to influences on; impacts their physical and mental health, including professional burnout, depression, and anxiety [1, 5, 15]. According to previous studies, the COVID-19 pandemic has made job burnout among healthcare practitioners one of the most prevalent and usually under-recognized mental health concerns [15]. 

Healthcare workers have been targets of stigma, which has led to harassment, stereotyping, discrimination, social isolation, and, in some cases, physical assault. People who undergo stigmatization may struggle with emotional disturbance, stress, depression, and anxiety [6]. During the post-pandemic period, it is anticipated that the prevalence of common psychiatric diseases and suicide will rise [6]. An increase in the prevalence of psychological distress, anxiety, depression, substance misuse disorders, suicide, and suicidal conduct has all been linked to economic challenges [6]. The factors that tend to determine post-economic recession mental health disorders include unemployment, unstable employment, a lower socioeconomic position, and pre-existing psychiatric conditions [6]. 

Due to the high burnout rate among healthcare professionals, mechanisms for local and national support have been started [5]. A previous study noted that inefficiencies in electronic medical records (EMRs), performance metrics, and limited professional autonomy often divert attention from initiatives to enhance patient care and provider well-being [12]. This study will help identify the burnout dental professionals and staff at an academic healthcare institution are facing. By analyzing, timely measures can be taken to reduce burnout and related mental health effects. Therefore, strategies such as mindfulness training, exercise, taking time off, and improving work-life balance are important for healthcare professionals [12]. The ability to deliver safe, high-quality, and patient-centered treatment is at risk in a healthcare population that is deteriorating in health and wellbeing [12]. 

Enhancing the availability of mental health resources like telepsychiatry, early diagnosis, treatment, psychological screening, and support, as well as implementing ongoing strategies to mitigate the impact of the economic crisis on mental well-being while also tackling stigma during the pandemic, are essential steps in addressing this problem [6]. Additionally, providing targeted support for specific groups, such as frontline healthcare professionals, when needed is crucial [6]. Time management, technology, telepresence, and other components of modern life assist us in determining our priorities and may enable prolonged clinical, educational, and research productivity without jeopardizing the qualities that render our humanity [12]. Efforts are underway nationwide in incorporating wellness programs in the workplace to address stress and poor job performance [13].

The purpose of this study is to assess the burnout rate among dental students, faculty, and staff post-COVID-19 at one academic healthcare institution, the University of California, San Francisco.

Research Question and Hypotheses:

1. How does burnout post COVID-19 burnout affect the productivity and motivation of the dental students, faculty and staff at UCSF?
  Null hypothesis: Burnout post COVID-19 burnout has no effect on the productivity and motivation of the dental students, faculty and staff at UCSF.
  Alternative hypothesis: Dental students, faculty, and staff have significantly impact on the productivity and motivation at UCSF post COVID-19 burnout.
2. Do factors like institutional and social support post COVID-19 affect the mental health of the dental students, faculty and staff at UCSF?
  Null hypothesis: Factors like institutional and social support post COVID-19 do not affect the mental health of the dental students, faculty and staff at UCSF.
  Alternative hypothesis: Factors like institutional and social support post COVID-19 affect the mental health of the dental students, faculty and staff at UCSF.

## Methods

This study was approved by the university’s institutional review board (IRB # 23-39814) and was deemed as exempt. A pilot study was performed to achieve power analysis of 109 surveys. A survey questionnaire was created through Qualtrics^®^ online survey tool consisted of questions related to demographics, physical and mental burnout. Principal and co-principal investigators distributed the survey to the dental students, residents, faculty, and staff at UCSF dental center via email. Participants were given 30 days.

The survey required 15 min to complete. Participants were given confirmation that their email would not be saved and anonymity assured. If any participant was not willing to take part in the survey process after reading the consent, they were free to do so. Any personal information was not used in any other condition. No financial compensation was given. The research project continued from August 2023 to October 2023. The data of all the survey form was transferred to an excel spreadsheet for accessible analysis. In August 2023, a literature review and IRB approval was obtained from the UCSF IRB committee. In September 2023, a survey design was finalized along with consent from the participants, and the survey was distributed. One of the survey questions included the frequency of having a challenging case. “Challenged” is defined as if the responders face any challenges in completing the case due to the complexity of clinical factors or patient factors that affect the well-being of respondents (dental students, residents, faculty, and staff). In October 2023, the quantitative analysis was done using Microsoft Excel (Microsoft 365), Microsoft Corporation, Redmond, WA, USA.

Inclusion criteria were all individuals over the age of 18 years at University of California San Francisco, School of Dentistry included dental students, residents, faculty, and staff. None of the students included in this study were in training prior to the COVID-19 pandemic, as the survey was conducted in September 2023, following the graduation of the Class of 2023 in May. The study included students from the 4-year DDS pathway who enrolled in 2020 and students from the 2-year International Dental Pathway (IDP) who enrolled in 2022; both cohorts expected to graduate in 2024. Students from a total of 70 predoctoral students from these two cohorts participated in the survey. There was no gender predilection in this study. Exclusion criteria were inadequate answers of the dental survey.

## Results

This study did not have any anticipated risks, discomfort, or inconvenience to the participants. This survey procedure did not harm the participants’ social, legal, physical, psychological, religious, and economic feelings or concerns.

A total of 109 dental faculty, residents, students, and staff at UCSF Dental Center completed the questionnaire from August to September 2023. We excluded one questionnaire due to no responses in any questions, yielding a response rate of 99.08%. A 21-item questionnaire was administered.

The descriptive demographic statistics included gender, age, job experience, specialty, and type of professionals were included. Gender consisted of 46 (42.59%) male, 61 (56.48%) female and 1 (0.93%) other. The age of the participants ranged from less than 30 years to more than 60 years old, with an average age of 27 years old (SD = 19.67).

The survey asked if dental professionals noticed any difference before and after the COVID-19 pandemic related to their wellbeing at work. Of the 108 participants, 60 (55.56%) indicated they felt a difference and 48 (44.44%) did not.

**Fig. 1:**
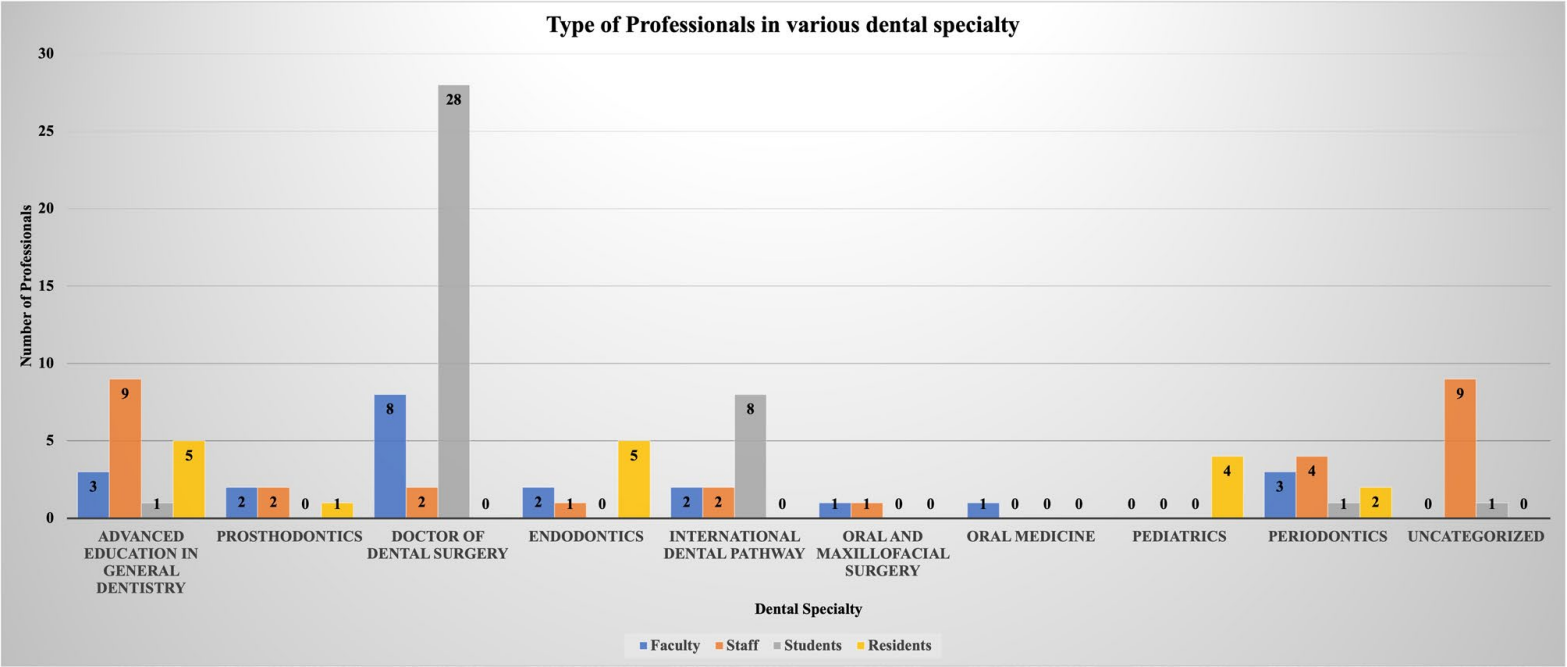
Types of Professionals in Various Dental Specialty

Figure 1 visualizes the type and number of professionals at UCSF. The number of individuals who completed the survey are as follows: faculty members, *n* = 22; staff members, *n* = 30; students, *n* = 39; and residents, *n* = 17 resulting in a total population of *n* = 108 individuals.

**Fig. 2:**
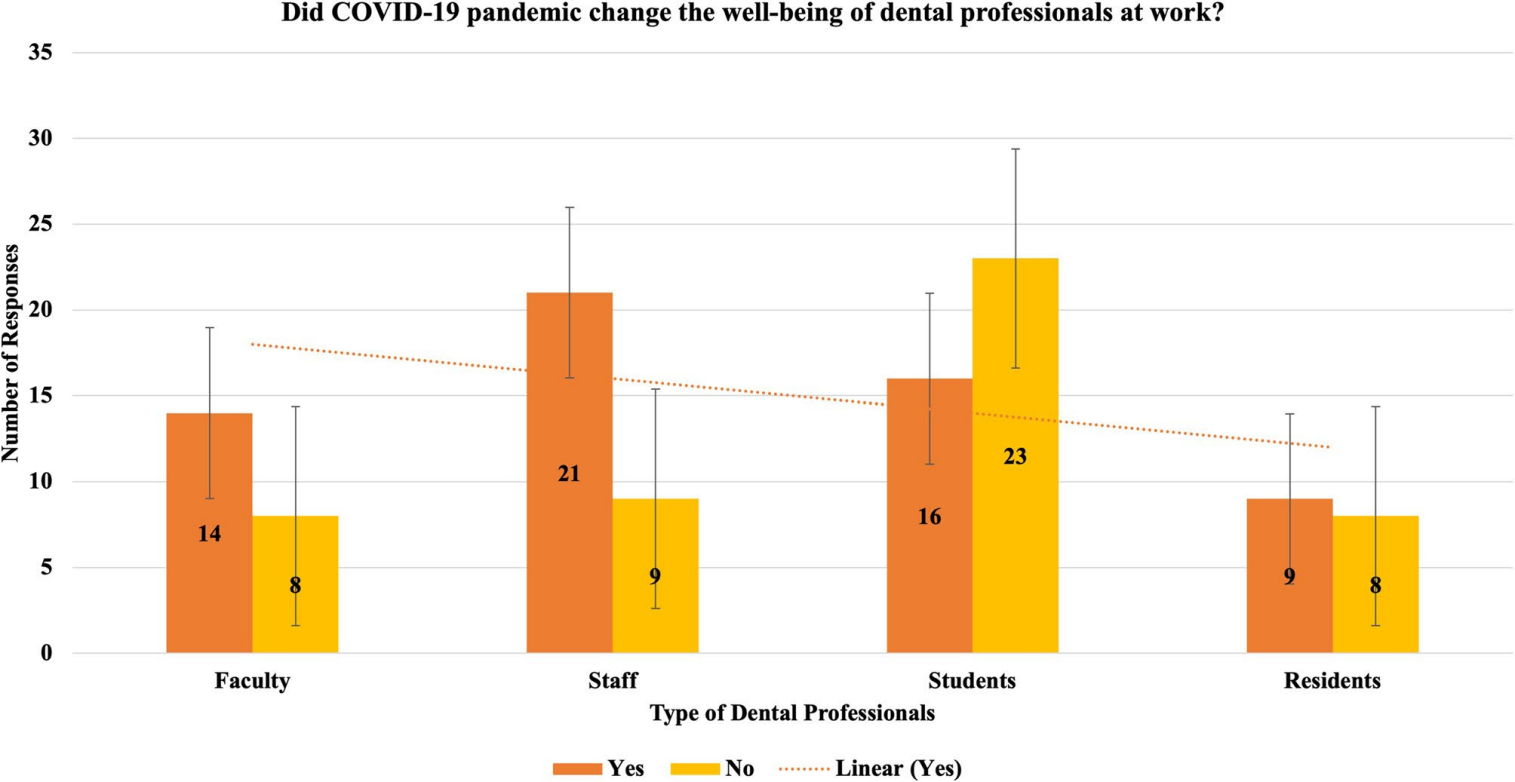
Did COVID-19 Pandemic change the Well-being of Dental Professionals at Work?

Figure 2 depicts the change in wellbeing at work for dental students, residents, faculty, and staff as a result of the COVID-19 pandemic. Dental students (*n* = 23) did not feel differences about their wellbeing at work where dental residents (*n* = 9), faculty (*n* = 14) and staff (*n* = 21) felt the difference. Of all the participants who responded yes, *n* = 44, the standard deviation was 4.97 and the participants who answered no, the standard deviation was 6.38. A trend with the participants who answered yes was shown with a linear dotted line.

Table 1 is about the institutional support the participants received regarding knowledge of COVID-19 diagnosis and treatment, emotional support for those who battled against COVID-19 and adequate personal protective equipment (PPE) and supply. Most participants rated institutional support as Good in all 3 categories.

**Table 1:** Institutional support during COVID-19 pandemic

Institutional Support during COVID-19 Pandemic (Participants *N* = 108) (%)					
	Poor	Fair	Average	Good	Excellent
Knowledge regarding COVID-19 Diagnosis and Treatment	5 (4.667)	14 (13.08)	30 (28.04)	41 (38.32)	17 (15.89)
Emotional support for appreciation for those battled against COVID-19	9 (8.41)	16 (14.95)	30 (28.04)	42 (39.25)	10 (9.35)
Adequate Equipment and supply (PPE)	4 (3.74)	6 (5.61)	22 (20.56)	43 (40.19)	32 (29.91)

Tables 2 and 3 gives information about (CBI) questions related to personal and work category. Out of the 108 respondents who completed the survey, 106 qualified as completed for the personal and work-related burnout scale with all burnout questions unanswered by two respondents. Personal and work-related burnout questions were categorized based on 5-point Likert scale. According to Table 2, among all personal CBI questions, an average of 11.78% professionals answered never, 20.44% answered rarely, 36.64% answered sometimes, 22.01% answered often and 9.12% professionals answered always. When comparing CB4 and CB6, distinct patterns emerge. CB4 reveals higher emotional strain, with only 29.25% answering never, while a combined 38.68% responded often or always, indicating significant psychological exhaustion. In contrast, CB6 reflects more physical resilience: 62.27% of participants answered never or rarely, and only 12.27% selected often or always. This suggests that while emotional overwhelm (CB4) is more prevalent, perceptions of physical vulnerability (CB6) are less common among participants. Table 3 shows an analysis where among all work-related CBI questions, average of 10% professionals answered very low/never, 21.4% answered low/seldom, 42.4% answered somewhat/sometimes, 21.8% answered high/often and 10.4% answered very high/always.

**Table 2:** Personal category of Copenhagen burnout (CB)

Personal category of Copenhagen Burnout (CB) (Participants *N* = 106) (%)					
	Never	Rarely	Sometimes	Often	Always
CB1: How often do you feel tired?	2 (1.89)	6 (5.66)	44 (41.51)	38 (35.85)	16 (15.09)
CB2: How often are you physically exhausted?	6 (5.56)	11 (10.38)	49 (46.23)	28 (26.42)	12 (11.32)
CB3: How often are you emotionally exhausted?	4 (3.77)	16 (15.09)	50 (47.17)	24 (22.64)	12 (11.32)
CB4: How often do you think: “I can’t take it anymore”?	31 (29.25)	34 (32.08)	23 (21.70)	14 (13.21)	4 (3.77)
CB5: How often do you feel worn out?	9 (8.49)	20 (18.87)	40 (37.74)	28 (26.42)	9 (8.49)
CB6: How often do you feel weak and susceptible to illness?	23 (21.70)	43 (40.57)	27 (25.47)	8 (7.55)	5 (4.72)

**Table 3:** Work-related category of Copenhagen burnout

Work-related category of Copenhagen Burnout					
	Very Low	Low	Somewhat	High	Very High
WR1: Is your work emotionally exhausting?	13 (12.26)	25 (23.58)	40 (37.74)	22 (20.75)	6 (5.66)
WR2: Do you feel burnt out because of your work?	13 (12.26)	20 (18.87)	43 (40.57)	18 (16.98)	12 (11.32)
WR3: Do you feel worn out at the end of the working day?	6 (5.66)	16 (15.09)	45 (42.45)	26 (24.53)	13 (12.26)
	Never	Seldom	Sometimes	Often	Always
WR4: Do you feel that every working hour is tiring for you?	14 (13.21)	34 (32.08)	36 (33.96)	17 (16.04)	5 (4.72)
WR5: Do you have enough energy for family and friends during leisure time?	4 (3.77)	12 (11.32)	48 (45.28)	26 (24.53)	16 (15.09)

Table 4 shows that the mean CBI subscale scores for this population were 17.37 for the personal subscale and 14.72 for the work-related subscale. However, after examining the overall proportion of dental students, residents, faculty, and staff, we found that they were experiencing moderate or higher burnout. Dental students, residents, faculty, and staff reported moderate burnout in 37.74% (*n* = 40 out of 106 cases), high burnout in 8.49% (*n* = 9) instances, and severe in 2 cases (1.89%), according to the personal subscale. Overall, 38.68% (*n* = 41 out of 106 instances) of dental students, residents, faculty, and staff reported moderate work-related burnout. One respondent had severe, while 15 (14.15%) dental students, residents, faculty, and staff reported having elevated levels of work-related burnout. A total of 55 (51.89%) respondents for personal and 49 (46.23%) respondents for work-related reported no or low levels of burnout.

**Table 4:** Prevalence of Copenhagen burnout inventory

Measure	Mean (Standard Deviation)	Prevalence cut-off *N* (%)
Copenhagen Burnout Inventory		
Personal burnout	17.37 (5.68)	No/Low (< 50) = 55 (51.89)
* N* = 106		Moderate (50–74) = 40 (37.74)
		High (75–99) = 9 (8.49)
		Severe (100) = 2 (1.89)
Work Burnout	14.72 (4.16)	No/Low (< 50) = 49 (46.23)
* N* = 106		Moderate (50–74) = 41 (38.68)
		High (75–99) = 15 (14.15)
		Severe (100) = 1 (0.94)

## Discussion

The present study investigates the burnout rate among dental students, faculty, and staff at the University of California, San Francisco (UCSF) in the aftermath of the COVID-19 pandemic. The study also assesses the level of institutional support provided to mitigate burnout and identify potential areas for improvement. The successful completion of the study will be used in reducing the incidence and prevalence of the burnout rate among the dental students, residents, faculty, and staff. An awareness will be raised not to neglect the mental health to reduce the anticipated risk to their mental and overall health.

The study’s demographic statistics revealed a relatively equal distribution between male and female participants, indicating a diverse sample. The average age of 27 years suggests that the study captured the experiences of dental professionals across various stages of their careers. This diverse representation enhances the generalizability of the findings and their applicability to a broader population of dental professionals.

The results indicated that more than half of the participants perceived a difference in their well-being at work following the COVID-19 pandemic. This finding aligns with previous research highlighting the impact of the pandemic on mental health and well-being across various professional domains [1, 8, 14]. The dental profession, in particular, experienced unique challenges due to increased infection control measures, changes in patient volume, predictable shortages of supplies, being unable to provide competent medical care, and the need to adapt to new protocols [15, 16]. Understanding the specific factors contributing to the perceived difference in well-being can inform targeted interventions to alleviate burnout and enhance the overall work environment.

According to the data in Fig. 2, dental students did not report a significant difference in their well-being at work before and after COVID-19 pandemic. The previous study suggests that dental students’ progress through their studies, they face increasing levels of workload stress, psychological strain, and burnout [17]. Here, the students from different classes responded, which included classes of 2023 and 2024. In contrast, dental residents, faculty, and staff indicated that they feel a difference in their well-being as a result of the pandemic. The distinction of well-being between dental students, residents, faculty and staff suggests that the impact of the pandemic on burnout and well-being might vary depending on the level of professional experience due to associated responsibilities. Dental residents, faculty, and staff, who often have more direct patient care responsibilities and administrative duties, may have faced greater challenges, and experienced a higher impact on their well-being [18]. This result obtained is inconsistent with a study conducted by Noori et al., which mentioned how work practice experience alone may not be a significant factor reducing burnout among dentists [11]. It is important to note that at the time the survey was conducted, the International Dental Pathway (IDP) program was two years in length; therefore, 40 students had not been enrolled during the COVID-19 pandemic. It includes 20 students from 2020 to 20 from 2021 of IDP. However, research by Zhang et al. did highlight other factors such as higher education, job responsibilities and challenging work positions also play a role in determining burnout levels [15]. 

The study assessed the level of institutional support received by dental professionals in three categories: COVID-19 diagnosis and treatment knowledge, emotional support, and personal protective equipment (PPE) and supplies. Most participants rated institutional support as “Good” in all three categories. This suggests that UCSF has implemented measures to provide essential resources and support to mitigate the negative impact of the pandemic. However, further exploration is needed to identify specific areas where additional support could be beneficial, as even with a “Good” rating, there may be room for improvement.

The Copenhagen Burnout Inventory (CBI) subscale scores revealed that both personal and work-related burnout were prevalent among the study participants. The moderate and high burnout rates in both categories underscore the urgent need to address this issue within the dental profession. The study’s findings align with existing literature which highlighted the elevated risk of burnout among healthcare professionals, particularly in high-demand and high-stress environments [12, 15]. The identification of burnout levels across distinct categories provides a clear understanding of the scope of the problem and supports the development of targeted interventions aimed at reducing burnout and promoting well-being.

This study contributes valuable insights into the burnout rate among dental professionals at UCSF following the COVID-19 pandemic. The findings emphasize the need for proactive measures to address burnout and support the well-being of dental students, faculty, and staff. The study also highlights the importance of institutional support and the potential impact it can have on mitigating burnout. By identifying areas for improvement and tailoring interventions to specific professional groups, dental institutions can create a healthier and more supportive work environment for their professionals. Reducing burnout enhances the well-being of dental professionals and positively impacts the quality of patient care and the overall dental healthcare system.

This study reveals distinct burnout profiles between dental professionals and dental students, reflecting the unique pressures inherent to each group. Dental professionals exhibited higher emotional exhaustion, likely from clinical workload, patient management, and administrative responsibilities. In contrast, students reported greater depersonalization and reduced personal accomplishment, suggesting stressors tied to academic performance, assessment demands, and career uncertainty.

While the pandemic offers important context for interpreting the experiences of dental professionals who faced shifting clinical protocols, patient safety concerns, and operational disruptions, the student data represent current burnout levels without causal ties to COVID-19. Explicitly acknowledging this distinction prevents overstating conclusions while preserving the comparative value of our findings.

Reframing the narrative, our emphasis is on contrasting the primary burnout drivers across the two cohorts: workplace stressors for professionals versus academic pressures for students. The pandemic serves as a lens only where directly relevant, such as for the professional group, ensuring that interpretations remain anchored to the sources of stress identified in our data rather than to a single external factor. This is consistent with evidence that burnout is multifactorial and context-specific, requiring separate intervention strategies for different career stages within dentistry.

Future studies should employ longitudinal designs to examine how burnout trajectories evolve with changing professional and academic demands, as well as to evaluate targeted interventions tailored to the differing needs of each population. Ultimately, these findings can serve as a framework for developing both institutional policies and curricular reforms aimed at mitigating burnout and supporting well-being across the dental career continuum.

## Conclusion

This study has highlighted a considerable level of burnout among students, faculty, and staff at UCSF post COVID-19 pandemic. These findings show the importance of continually monitoring the mental well-being of these individuals. Further exploration into the factors contributing to this burnout and strategies to alleviate it, would be valuable for institutions aiming to provide effective support to their students and employees as they navigate the challenges posed by the results of the pandemic.

## Supplementary Information

Supplementary Material 1.

## Data Availability

The data analyzed during the current study is available from the corresponding author on reasonable request.

## Abbreviations

UCSF: University of California, San Francisco
CBI: Copenhagen Burnout Inventory
PPE: Personal Protective Equipment

## References

[1] Bai X, Wan Z, Tang J, Zhang D, Shen K, Wu X, Qiao L, et al. The prevalence of burnout among pulmonologists or respiratory therapists pre-and post-COVID-19: a systematic review and meta-analysis. Ann Med. 2023;55(1):2234392. 37459584 10.1080/07853890.2023.2234392 PMC10353333

[2] Che L, Ma S, Zhang YL, Huang Y. Burnout among Chinese anesthesiologists after the COVID-19 pandemic peak: a National survey. Anesth Analgesia. 2023;137(2):392–8. 10.1213/ANE.0000000000006298 PMC10319244 36729947

[3] Fiabane E, Margheritti S, Nicolò E, Aiello S, Magnone M, Miglioretti P, Gabanelli, Giorgi I. Prevalence and determinants of Italian physicians’ burnout in the post-COVID-19 era. Int Arch Occup Environ Health. 2023;96(3):377–87. 36335513 10.1007/s00420-022-01929-6 PMC9638242

[4] Ghoneim A, Parbhakar KK, Farmer J, Quinonez C. Healthy and respectful workplaces: the experiences of dental hygienists in Canada. JDR Clin Translational Res. 2022;7(2):194–204. 10.1177/23800844211001827 33754872

[5] Kadhum M, Farrell S, Hussain R, Molodynski A. Mental wellbeing and burnout in surgical trainees: implications for the post-COVID-19 era. J Br Surg. 2020;107(8):e264–264. 10.1002/bjs.11726 PMC7283838 32463518

[6] Kathirvel N. Post COVID-19 pandemic mental health challenges. Asian J Psychiatry. 2020;53:102430. 10.1016/j.ajp.2020.102430 PMC7507979 33264840

[7] Kirkpatrick H, Wasfie T, Laykova A, Barber K, Hella J, Vogel M. Emotional intelligence, burnout, and wellbeing among residents as a result of the COVID-19 pandemic. Am Surg. 2022;88(8):1856–60. 35393863 10.1177/00031348221086804 PMC9001052

[8] Lee J. Cho, and Sung rae shin. Nursing strategies for the post-COVID‐19 era. Int Nurs Rev. 2021;68(2):149–52. 33406273 10.1111/inr.12653 PMC9292181

[9] Liu C, Yue C, Liu L, Liu T, Wang X, Hou Y, Gao S. Mediating role of perceived social support in the relationship between perceived stress and job burnout among midwives in the post-COVID‐19 era. Nursing Open. 2023;10(2):479–487. 10.1002/nop2.1313 PMC9834135 35964290

[10] Margheritti S, Giorgi I, Magnone S, Miglioretti M, Elena Fiabane. Physicians’ turnover intention during the Post–COVID-19 era: risk and protective factors. J Occup Environ Med. 2023;65(10):e631–5. 37442758 10.1097/JOM.0000000000002922

[11] Noori F, Kazemeini S-K, Owlia F. Determination of professional job burnout and temperament (Mizaj) from the viewpoint of Traditional Persian Medicine and work-related variables among Iranian dentists: a cross-sectional study. BMC Psychol. 2022;10(1):94. 35395954 10.1186/s40359-022-00803-x PMC8994344

[12] Romanelli J, Gee D, Mellinger JD, Alseidi A, Bittner JG, Auyang E, Asbun H. Feldman, and SAGES reimagining the practice of surgery task force. The COVID-19 reset: lessons from the pandemic on burnout and the practice of surgery. Surg Endosc. 2020;34:5201–7. 33051763 10.1007/s00464-020-08072-8 PMC7552950

[13] Altman J, Firebaugh CM, Morgan SM, Epstein M. Perceived Workplace Support for Employee Participation in Workplace Wellness Programs: A Brief Report. Merits. 2023;3(3):494–503.

[14] Živanović D, Javorac J, Stojkov S, Jevtić M, Knežević J, Blanuša J, Štimac Grbić D, KUSTURICA M. N. Jovanović lješković, and N. Todorović. The COVID-19 pandemic-related psychological distress and job burnout among Serbian pharmacy practitioners: a cross-sectional online study. Eur Rev Med Pharmacol Sci. 2022;26:7. 10.26355/eurrev_202204_28502 35442480

[15] Zhang Y, Li Y, Lu HL, Yang J, Wang Y, Liu J, Pu L, Liu. Xiaogang zhong, and Jin xin. Occupational differences in psychological distress between Chinese dentists and dental nurses. Front Psychol. 2022;13:923626. 35846642 10.3389/fpsyg.2022.923626 PMC9285401

[16] Zhou T, Xu C, Wang C, Sha S, Wang Z, Zhou Y, Zhang X, et al. Burnout and well-being of healthcare workers in the post-pandemic period of COVID-19: a perspective from the job demands-resources model. BMC Health Serv Res. 2022;22(1):284. 35236354 10.1186/s12913-022-07608-z PMC8888816

[17] Gil Y, Min JS, Hong JL, Ban J-S, Kwon, Jae-Il L. Dental students’ perception of their educational environment in relation to their satisfaction with dentistry major: a cross-sectional study. BMC Med Educ. 2023;23(1):508. 37461010 10.1186/s12909-023-04485-w PMC10351200

[18] National Institutes of Health. Oral health in America: advances and challenges. Bethesda, MD: US Department of Health and Human Services, National Institutes of Health, National Institute of Dental and Craniofacial Research. 2021.

