# Supplementary material for: Burnout rate among dental professionals post COVID-19 at one academic dental institution
Source: BMC Med Educ. 2025 Sep 25;25:1261. doi: 10.1186/s12909-025-07841-0 (PMC12465423; doi:10.1186/s12909-025-07841-0)
Supplement: Supplementary file 1 — Supplementary Material 1. [file 12909_2025_7841_MOESM1_ESM.docx]

**Survey questionnaire :**

This questionnaire is for the betterment of your mental and overall health. You do not have to indicate your name anywhere. Please be honest so that we can help you our best. Thank you.

| **Demographic characteristics** | | |
| --- | --- | --- |
| **Gender** | Male |  |
|  | Female |  |
|  | Other |  |
| **Age** | <30 years |  |
|  | 31–40 years |  |
|  | 41-50 years |  |
|  | 51-60 years |  |
|  | >60 years |  |
| **Job Experience** | <5 years |  |
|  | 5-15 years |  |
|  | 16-25 years |  |
|  | >25 years |  |
| **Specialty** | Advanced education in General dentistry |  |
|  | Periodontics |  |
|  | Prosthodontics |  |
|  | Oral and Maxillofacial Surgery |  |
|  | Pediatrics |  |
|  | Oral Medicine |  |
|  | Endodontics |  |
|  | Doctor of Dental Surgery |  |
|  | International Dental Pathway |  |
| **Type of Professionals** | Students |  |
|  | Residents |  |
|  | Faculty |  |
|  | Staff |  |

| **Related to wellbeing affected due to pandemic and frequency of challenging case** | | |
| --- | --- | --- |
| **Pandemic affected wellbeing at work?** | Yes |  |
|  | No |  |
| **How often do you manage the challenging case** | Almost never |  |
|  | Once a month |  |
|  | Once a week |  |
|  | 2-3 times a week |  |
|  | Almost daily |  |

How would you rate your Institutional support during COVD-19 pandemic?

|  | Poor | Fair | Average | Good | Excellent |
| --- | --- | --- | --- | --- | --- |
| Knowledge regarding COVID-19 diagnosis and treatment needed | ○ | ○ | ○ | ○ | ○ |
| Emotional support for appreciation for those who battled against COVID-19 | ○ | ○ | ○ | ○ | ○ |
| Adequate Equipment and supply (PPE) | ○ | ○ | ○ | ○ | ○ |

*Categories of burnout and distress*

Copenhagen Burnout Inventory: rate each statement on a five-point Likert scale (from never to always)

1. Personal

|  | Never | Rarely | Sometimes | Often | Always |
| --- | --- | --- | --- | --- | --- |
| How often do you feel tired? | ○ | ○ | ○ | ○ | ○ |
| How often are you physically exhausted? | ○ | ○ | ○ | ○ | ○ |
| How often are you emotionally exhausted? | ○ | ○ | ○ | ○ | ○ |
| How often do you think: “I can’t take it anymore”? | ○ | ○ | ○ | ○ | ○ |
| How often do you feel worn out? | ○ | ○ | ○ | ○ | ○ |
| How often do you feel weak and susceptible to illness? | ○ | ○ | ○ | ○ | ○ |

Scoring: Always: 100. Often: 75. Sometimes: 50. Rarely: 25. Never/almost never: 0.

Total score on the scale is the average of the scores on the items.

If less than three questions have been answered, the respondent is classified as non-responder.

1. Work-related

|  | Very low | Low | Somewhat | High | Very High |
| --- | --- | --- | --- | --- | --- |
| Is your work emotionally exhausting? | ○ | ○ | ○ | ○ | ○ |
| Do you feel burnt out because of your work? | ○ | ○ | ○ | ○ | ○ |
| Do you feel worn out at the end of the working day? | ○ | ○ | ○ | ○ | ○ |

|  | Never | Seldom | Sometimes | Often | Always |
| --- | --- | --- | --- | --- | --- |
| Do you feel that every working hour is tiring for you? | ○ | ○ | ○ | ○ | ○ |
| Do you have enough energy for family and friends during leisure time? | ○ | ○ | ○ | ○ | ○ |

Response categories:

First three questions: To a very high degree, To a high degree, Somewhat, To a low degree, To a very low degree.

Last two questions: Always, Often, Sometimes, Seldom, Never/almost never. Reversed score for last question.

Scoring same as the first scale. If less than three questions have been answered, the respondent is classified as non-responder.
